# Concurrent Spinal Dural Arteriovenous Fistula and Varicella-Zoster Virus Meningoencephalitis Unmasked by Corticosteroid-Associated Deterioration: A Case Report on the Diagnostic Value of Serial mNGS

**DOI:** 10.2174/0115734056496224260602072444

**Published:** 2026-06-04

**Authors:** Mengdi Hao, Yuhui Sha, Jun Gao, Jingwen Niu, Yan Xu

**Affiliations:** 1 Department of Neurology, Peking Union Medical College Hospital, Chinese Academy of Medical Sciences and Peking Union Medical College, Beijing, 100730, China; 2 Department of Neurosurgery, Peking Union Medical College Hospital, Chinese Academy of Medical Sciences and Peking Union Medical College, Beijing, 100730, China

**Keywords:** Spinal dural arteriovenous fistula, Digital subtraction angiography, Varicella-zoster virus, Metagenomic next-generation sequencing, Ventricular drainage, Angiography

## Abstract

**Background:**

Concurrent spinal dural arteriovenous fistula (SDAVF) and varicella-zoster virus (VZV) meningoencephalitis are exceptionally rare, and overlapping features can delay diagnosis. This case adds to the literature by illustrating how corticosteroid exposure before exclusion of vascular and infectious mimics may be followed by neurological deterioration, and by emphasizing the diagnostic value of serial metagenomic next-generation sequencing (mNGS).

**Case Presentation:**

A 48-year-old man developed insidious bilateral lower-limb weakness that progressed to numbness, sphincter dysfunction, and near-paralysis. Initial spinal magnetic resonance imaging showed diffuse thoracolumbar cord lesions; cerebrospinal fluid studies were mildly inflammatory, and myelitis was suspected. He received methylprednisolone pulse therapy followed by oral corticosteroids without improvement. One month later, he presented with fever, severe headache, vomiting, worsening paralysis, and altered mental status. Cerebrospinal fluid demonstrated marked pleocytosis, hypoglycorrhachia, and elevated protein, and mNGS detected abundant VZV sequences. Brain imaging showed hydrocephalus, meningeal enhancement, multifocal ischemic lesions, and intracranial arterial stenoses, consistent with VZV meningoencephalitis and vasculopathy. After external ventricular drainage, intravenous acyclovir, dexamethasone for cerebral edema, and empirical anti-tuberculosis therapy, serial mNGS showed a reduced VZV burden. Repeat spinal imaging revealed tortuous perimedullary vessels and hemosiderin deposition, and angiography confirmed SDAVF from the left T10 intercostal artery. The fistula was coagulated. At 12-month follow-up, he regained slight right-leg movement and partial sensory recovery above L1.

**Conclusion:**

Progressive myelopathy with atypical inflammatory features should prompt vascular evaluation and pathogen testing. Serial mNGS can identify coexisting infection, guide therapy, and help avoid hazardous empirical corticosteroid use when the diagnosis remains uncertain.

## 1 INTRODUCTION

Spinal dural arteriovenous fistula (SDAVF) is a rare cause of progressive spinal cord lesions, with an annual incidence of 5 to 10 cases per million people [1]. Due to its diverse clinical manifestations and lack of specificity, misdiagnosis and missed diagnosis frequently occur, posing a serious threat to patients' health. Definitive diagnosis relies on digital subtraction angiography (DSA).

Varicella-zoster virus (VZV) often poses challenges for clinical diagnosis due to its protean manifestations and overlap with multiple inflammatory and infectious disorders [2]. Although polymerase chain reaction (PCR) is the gold standard for detecting VZV, false-negative results may occur due to primer specificity [3]. According to previous studies, metagenomic next-generation sequencing (mNGS) technology offers unique advantages for identifying various viral meningoencephalitis, including high sensitivity, rapid testing, and broad-spectrum detection [3].

Although previous studies have reported the application of mNGS in VZV identification [2, 3], the coexistence of SDAVF with VZV meningoencephalitis in this case is extremely rare. Furthermore, the diagnostic value of mNGS in immunocompromised patients with deteriorating neurological function remains understudied. Therefore, this case report not only expands the clinical spectrum of SDAVF but also underscores the critical role of mNGS in diagnosing complex neurological infections, offering significant complementary value to the existing literature. This case report was prepared in accordance with the CARE guidelines.

## 2 CASE REPORT

**Fig. (1):**
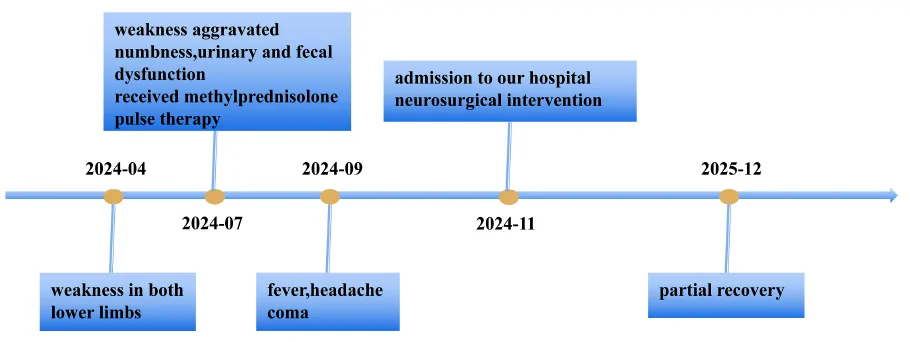
Patient treatment timeline flowchart.

**Fig. (2):**
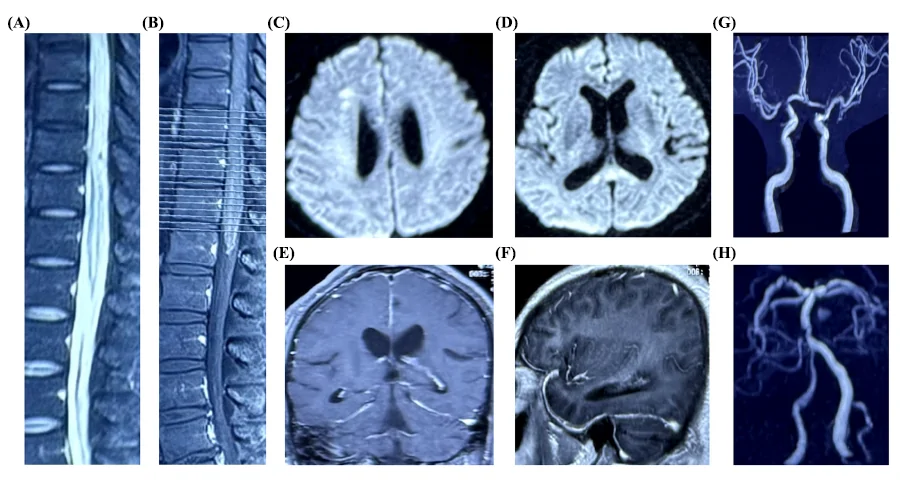
Initial spinal and head MRI of the patient. **A**. MRI approximately four days after the onset of worsening weakness, numbness, and dysuria revealed extensive T2 hyperintensity in the sagittal view of the thoracic spinal cord; **B**. On sagittal T1-enhanced imaging; **C**,**D**. The thoracic spinal cord was extensively enhanced. DWI sequences revealed multiple patches of high signal in the right periventricular region and the splenium of the corpus callosum; **E**,**F**. Enhancement sequences revealed extensive enhancement of the dura mater; **G**,**H**. MRA demonstrated severe and multifocal segmental stenosis of the right vertebral artery, basilar artery, and middle cerebral arteries. MRI, magnetic resonance imaging; DWI, diffusion weighted imaging; MRA, magnetic resonance angiography. Scale bars = 10 mm.

In April 2024, a 48-year-old man experienced insidious-onset weakness in both lower limbs, initially causing difficulty climbing stairs (Fig. **1**). Over the subsequent three months, his symptoms remained stable, and he could walk freely. Subsequently, in July 2024, the weakness rapidly aggravated, and he developed numbness, urinary and fecal dysfunction, and almost complete loss of lower limb mobility over three days. Neurological examination at a local hospital revealed grade II muscle strength in both lower limbs, bilateral positive pathological reflexes, and hypoalgesia below the T12 dermatome level. Contrast-enhanced magnetic resonance imaging (MRI) revealed diffuse thoracolumbar spinal cord lesions with enhancement (Fig. **2A**, **B**). Initial spinal MRI showed diffuse T2 hyperintensity and enhancement in the thoracolumbar spinal cord, but no obvious flow voids or abnormal perimedullary vascular structures were identified. Cerebrospinal fluid (CSF) analysis revealed an opening pressure of 150 mmH_2_O, a white blood cell count (WBC) of 9 × 10^6^/L (55% mononuclear cells), a glucose level of 4.7 mmol/L, a chloride level of 126 mmol/L, and a protein level of 0.47 g/L, with no oligoclonal bands detected. The patient was diagnosed with myelitis and received methylprednisolone pulse therapy followed by oral corticosteroids. However, his symptoms did not improve significantly. At discharge, he remained unable to move his lower limbs. Following one month of rehabilitation, he regained slight antigravity movement and was able to actively lift his legs off the bed.

One month later (September 2024), the patient developed fever, severe headache with vomiting, worsening lower limb weakness, and an altered mental status. A repeat lumbar puncture revealed an opening pressure of 340 mmH_2_O, with yellow and cloudy CSF, a WBC count of 383 × 10^6^/L, a protein level of 7.53 g/L, a glucose level of 1.82 mmol/L, and a chloride level of 10^9^ mmol/L. A total of 3,499 VZV sequences were also detected by mNGS. The patient had no history of varicella-zoster virus vaccination prior to the onset of symptoms. CSF acid-fast staining, Xpert *Mycobacterium tuberculosis (*MTB*)*/rifampicin assays, and MTB culture were all negative. Head computed tomography (CT) revealed hydrocephalus. Due to severely elevated intracranial pressure, the patient experienced two episodes of brain herniation, prompting urgent placement of an external ventricular drain. Contrast-enhanced brain MRI revealed multiple diffusion-restricted lesions involving the right centrum semiovale, a right periventricular region, an internal watershed region, and the splenium of the corpus callosum, with diffuse meningeal enhancement (Fig. **2C**-**F**). Furthermore, head CT identified local subarachnoid hemorrhage while magnetic resonance angiography (MRA) revealed multiple segmental stenoses of the intracranial arteries (Fig. **2G**, **H**), indicating VZV-related vasculitis. The patient was initiated on intravenous acyclovir (0.75 g every 8 hours) and dexamethasone therapy. Furthermore, given the severe meningeal inflammation, CNS tuberculosis (TB) infection could not be excluded. Thus, empirical four-drug anti-tuberculosis therapy was commenced. The patient’s fever resolved, accompanied by concurrent neurological improvement.

**Fig. (3):**
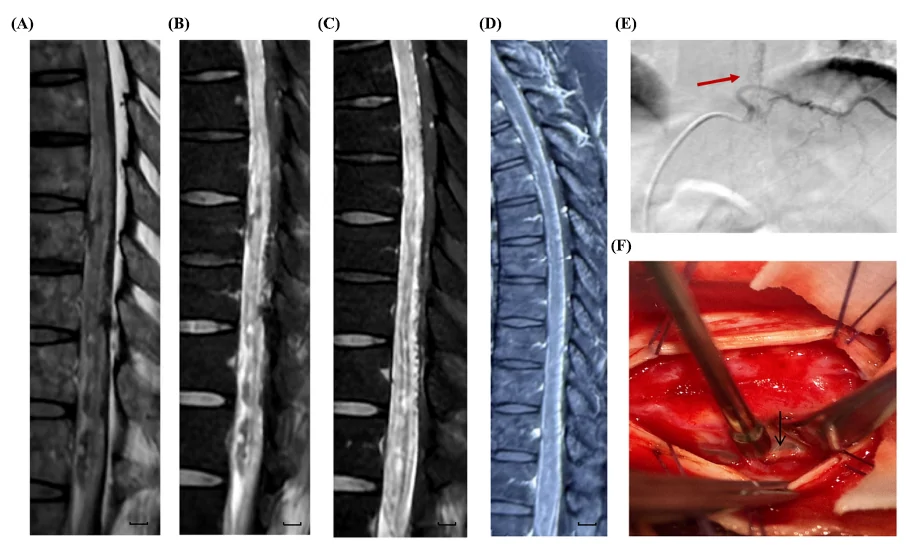
Spinal cord MRI and spinal angiography. **A**. MRI of the thoracic spine revealed multiple patchy high T2WI signals in the thoracic spinal cord and hemosiderin deposition in the dorsal spinal cord; **B**. Multiple curved vascular structures around the spine on fat-suppression sequence; **C**. Irregular thickening and enhancement of the spinal meninges; **D**. On angiography of the left T10 intercostal artery; **E**. The dural veins were early and tortuous and dilated (red arrow); **F**. Intraoperative findings of arterialized veins proximal to the fistula (white arrow). MRI, magnetic resonance imaging; T2WI, T2-weighted imaging. Scale bars = 10 mm.

Upon admission to our hospital, the patient was alert but exhibited impaired orientation and cognitive decline. Neurological examination revealed complete paralysis (grade 0) of both lower limbs, an absence of pathological reflexes, hypalgesia below T6, and a diminished vibration sensation below T12. Repeat lumbar puncture revealed an opening pressure of 165 mmH_2_O and a lymphocytic-predominant pleocytosis, with a WBC count of 57 × 10^6^/L, protein of 1.87 g/L, glucose of 3.9 mmol/L, chloride of 119 mmol/L, and 39 VZV sequences detected by mNGS. Spinal MRI demonstrated multiple patchy lesions in the thoracic spinal cord, irregular meningeal thickening and enhancement, multiple tortuous perimedullary vascular structures, and the deposition of hemosiderin (Fig. **3A**-**D**).

Spinal angiography confirmed an early and tortuous draining vein originating from the left T10 intercostal artery, consistent with SDAVF (Fig. **3E**). Given the persistent lower limb paralysis, the presence of tortuous perimedullary vascular structures and hemosiderin deposition on repeat spinal MRI, and the poor response to prior therapies, spinal angiography was performed to evaluate for a possible spinal vascular malformation, which confirmed SDAVF. The patient subsequently underwent neurosurgical intervention, during which a fistulous connection between the radiculomedullary artery and vein was identified and successfully coagulated (Fig. **3F**). Postoperatively, the patient exhibited occasional involuntary movements in the right foot, although his overall lower limb strength remained at grade 0, with a sensory level at T8. The patient completed a 1.5-month course of intravenous acyclovir (0.75 g q8h) for VZV infection, alongside a full six-month course of empirical anti-tuberculosis therapy. At the 12-month follow-up, the patient had regained slight movement in the right leg with partial recovery of sensation above the L1 dermatome.

## 3 DISCUSSION

This report presents a unique case of a middle-aged man who was initially diagnosed with myelitis, whose condition acutely worsened after corticosteroid therapy was administered before exclusion of vascular and infectious mimics, due to concurrent VZV meningoencephalitis. Subsequent re-evaluation ultimately revealed an underlying SDAVF.

SDAVF is a rare but critical spinal vascular disorder that is often misdiagnosed as polyradiculopathy or anterior horn cell disease. This condition results from an abnormal connection between a radicular artery and vein, leading to venous congestion and ischemic spinal cord damage [4, 5]. The hallmark feature of SDAVF is progressive myelopathy. MRI is the initial diagnostic modality, while DSA remains the gold standard for definitive diagnosis. Characteristic MRI findings include spinal cord swelling with central T2 hyperintensity and voids in dorsal perispinal flow [6].

SDAVF is frequently misdiagnosed as myelitis due to overlapping clinical features. A poor response to corticosteroids should prompt consideration of vascular pathology, and MRI should be scrutinized for abnormal vascular signals [7]. While the temporal association between corticosteroid administration and clinical worsening is noteworthy, it is acknowledged that this relationship is not necessarily causal, as the deterioration could also be attributable to the natural course of VZV meningoencephalitis. Corticosteroids may have contributed to immunosuppression and viral reactivation, but alternative explanations, including the inherent severity of VZV infection, cannot be excluded. Notably, the clinical course in this case strongly suggests a causal hierarchy: the insidious onset of lower limb weakness in April 2024, followed by progressive myelopathy with characteristic MRI findings (flow voids and hemosiderin deposition), points to SDAVF as the primary chronic pathology. The subsequent administration of high-dose corticosteroids likely induced immunosuppression, leading to reactivation of latent VZV and the development of secondary meningoencephalitis and vasculopathy. This temporal sequence underscores the importance of considering SDAVF as the underlying etiology in patients with progressive myelopathy and highlights the risk of corticosteroid therapy in unmasking occult infections.

The neurological manifestations of VZV infection, including encephalitis, myelitis, and vasculopathy, can occur without a rash, a condition known as zoster sine herpete. The risk of severe VZV-related neurological complications is significantly elevated in immunocompromised individuals due to the ability of this virus to effectively evade the immune system, mandating careful risk-benefit analysis prior to initiating high-dose immunosuppressive regimens [8, 9]. In this case, dexamethasone was used adjunctively to control life-threatening cerebral edema and inflammatory complications, balancing the risk of exacerbating viral replication with the urgent need to reduce intracranial pressure. Empirical anti-tuberculous therapy was maintained because severe meningeal inflammation, hydrocephalus, and high CSF protein levels raised persistent concern for partially treated CNS tuberculosis despite repeated negative microbiological assays. The decision was made after careful risk-benefit analysis, weighing the potential for irreversible neurological damage from untreated TB against the risks of hepatotoxicity and drug interactions, which were monitored closely throughout the six-month course. Previous studies have found that approximately 60% of acute meningoencephalitis cases remain undiagnosed after PCR testing [2]. In a previous study comparing diagnostic methods for VZV neurological infections, the detection rate of mNGS was significantly higher than that of PCR (100% *vs*. 66.7%, *p* < 0.05) and serum Human Immunoglobulin M antibodies (100% *vs*. 68.8%, *p* < 0.05) [10]. Additionally, mNGS can reduce diagnostic time to within three days, highlighting its significant advantages in managing complex and atypical cases of neurological infections [2, 3].

Venous congestion secondary to SDAVF can cause spinal cord edema, meningeal enhancement, and even inflammatory changes on MRI, which may mimic infectious myelitis or meningitis. In this case, the initial spinal MRI findings, including diffuse T2 hyperintensity and enhancement, could partly be attributed to SDAVF-induced venous hypertension. However, the subsequent development of fever, headache, altered mental status, markedly elevated CSF white blood cell count and protein level, and detection of VZV by mNGS confirmed a concurrent infectious etiology. The presence of hydrocephalus and vasculopathy further supported VZV meningoencephalitis rather than SDAVF alone. Thus, while SDAVF-related inflammation may have contributed to the initial imaging abnormalities, the clinical deterioration and laboratory findings were driven by superimposed VZV infection.

The timing of SDAVF treatment in this case was critically influenced by the concurrent VZV meningoencephalitis and life-threatening intracranial hypertension. Due to the urgent need for external ventricular drainage and antiviral therapy to control cerebral edema and herniation, the surgical intervention for SDAVF was deferred until the patient’s neurological and infectious status stabilized. This decision was made after multidisciplinary consultation, balancing the risks of delayed SDAVF treatment against the immediate threat of brain herniation. Postoperative recovery of lower limb function was limited, likely reflecting the prolonged venous congestion and irreversible spinal cord damage prior to surgical correction. This case underscores that early DSA evaluation and prompt SDAVF treatment should be prioritized when feasible, but may need to be carefully sequenced in the presence of concurrent life-threatening infections.

Compared to previous studies, this case report presents a unique instance of coexisting SDAVF and VZV meningoencephalitis. By detailing the diagnostic process, treatment response, and final confirmation of this case, the report aims to enhance clinicians' awareness of such rare and complex conditions. Learning points are described in Table **1**. This will facilitate early and accurate diagnosis, enabling the development of more effective treatment strategies to improve patient prognosis and quality of life.

Some clinical features, such as altered mental status, headache, and imaging findings of hydrocephalus and meningeal enhancement, could theoretically be attributed solely to vascular pathology, such as VZV-related vasculitis or secondary ischemic changes from SDAVF-induced venous congestion. However, the detection of VZV sequences by mNGS in the CSF, along with the presence of pleocytosis and elevated protein levels, strongly supports an infectious component. Nevertheless, the generalizability of this case is limited; in this complex case, distinction between purely vascular and infectious etiologies remains challenging in this complex case, and the contribution of each cannot be fully disentangled.

## CONCLUSION

This case highlights the importance of early DSA evaluation in patients with unexplained myelopathy, particularly in middle-aged men. Furthermore, concurrent VZV infection should be suspected in patients who develop fever, headache, and cognitive impairment following immunosuppressive therapy. mNGS is a valuable tool for the identification of pathogens. Timely SDAVF diagnosis and intervention are critical for improving outcomes, while appropriate antiviral therapy is essential for managing VZV-related complications.

## Figures and Tables

**Table 1:** Learning points.

No.	Key take-home messages
1	In patients with progressive myelopathy, especially middle-aged men, SDAVF must be considered in the differential diagnosis, and DSA should be performed promptly if clinical or radiological features are suggestive.
2	High-dose corticosteroid therapy in the context of an undiagnosed SDAVF may precipitate immunosuppression and lead to the reactivation of latent infections such as VZV, resulting in severe complications like meningoencephalitis and vasculitis.
3	mNGS of cerebrospinal fluid is a powerful diagnostic tool for identifying pathogens in complex or atypical central nervous system infections, particularly when conventional tests are negative or clinical suspicion remains high.
4	The management of complex neurological cases with overlapping vascular and infectious pathologies requires a multidisciplinary approach, balancing targeted interventions (*e.g*., fistula occlusion, antiviral therapy) with supportive care, while carefully weighing the risks and benefits of empirical treatments.
5	A detailed temporal analysis of symptom progression, treatment responses, and diagnostic findings is crucial for unraveling the sequence of events in complex cases and for attributing clinical deterioration to specific etiologies or interventions.

**Abbreviations:** SDAVF: spinal dural arteriovenous fistula; DSA: digital subtraction angiography; VZV: varicella-zoster virus; mNGS: metagenomic next-generation sequencing.

## Data Availability

The dataset used for the current case report is available from the corresponding authors [Y.X] and [J.N.] on reasonable request.

## LIST OF ABBREVIATIONS

CARE: Case Report guidelines
CNS: Central nervous system
CSF: Cerebrospinal fluid
CT: Computed tomography
DSA: Digital subtraction angiography
DWI: Diffusion-weighted imaging
MRA: Magnetic resonance angiography
MRI: Magnetic resonance imaging
MTB: Mycobacterium tuberculosis
mNGS: Metagenomic next-generation sequencing
PCR: Polymerase chain reaction
SDAVF: Spinal dural arteriovenous fistula
T2WI: T2-weighted imaging
TB: Tuberculosis
VZV: Varicella-zoster virus
WBC: White blood cell

## STANDARDS OF REPORTING

CARE guidelines were followed.
